# Myeloid Responses to Extracellular Vesicles in Health and Disease

**DOI:** 10.3389/fimmu.2022.818538

**Published:** 2022-03-07

**Authors:** Priya Makhijani, Tracy L. McGaha

**Affiliations:** ^1^ Department of Immunology, University of Toronto, Toronto, ON, Canada; ^2^ Tumor Immunotherapy Program, Princess Margaret Cancer Center, University Health Network, Toronto, ON, Canada

**Keywords:** macrophage, extracellular vesicle, cancer, autoimmune disease, inflammation

## Abstract

Extracellular vesicles are mediators of cell-cell communication playing a key role in both steady-state and disease conditions. Extracellular vesicles carry diverse donor-derived cargos, including DNA, RNA, proteins, and lipids that induce a complex network of signals in recipient cells. Due to their ability to capture particulate matter and/or capacity to polarize and orchestrate tissue responses, myeloid immune cells (e.g., dendritic cells, macrophages, etc.) rapidly respond to extracellular vesicles, driving local and systemic effects. In cancer, myeloid-extracellular vesicle communication contributes to chronic inflammation, self-tolerance, and therapeutic resistance while in autoimmune disease, extracellular vesicles support inflammation and tissue destruction. Here, we review cellular mechanisms by which extracellular vesicles modulate myeloid immunity in cancer and autoimmune disease, highlighting some contradictory results and outstanding questions. We will also summarize how understanding of extracellular vesicle biology is being utilized for novel therapeutic and diagnostic applications.

## 1 Introduction

Extracellular vesicles (EVs) are phospholipid bilayer-bound particles shown to be produced by all tested cell types. These cell-derived vesicles are released into the extracellular space accumulating in tissue, circulation and other fluids (1). Studies on EV cargo, subtype heterogeneity, and cell responses have shown they play diverse roles in health and disease. EV cargos include protein, DNA, mRNA, non-coding RNA and lipids from the donor cell, with specific cargo enrichment depending on cell status and EV subtype (2). The three most examined subtypes of EVs are apoptotic bodies (ABs), ectosomes and exosomes, though novel EV subtypes are still being identified. EVs can elicit potent autocrine, paracrine, and systemic responses in many cell types including in macrophages, endothelial cell, and lymphocytes (3). EV responses have been shown to regulate physiological processes including inflammation and tissue regeneration (3). Therefore, EVs are increasingly recognized as key mediators of intra- and inter-cellular communication, akin to cytokine signals. However, unlike most cytokines, EVs are highly stable and carry diverse cargo allowing EVs to drive complex responses both locally and at distant sites. Responses to EVs are dictated by many factors including capture mechanisms and physiological context. EVs are internalized *via* multiple mechanisms including receptor-mediated and lipid-raft mediated endocytosis, phagocytosis, and pinocytosis (3). Depending on route of internalization, EV cargo is delivered to different cellular compartments driving rapid recycling, interaction with endosomal receptors, and/or antigen presentation leading to different responses (4). EV responses *via* cell surface receptor interactions have also been reported, especially in cells that are poor at EV capture. For example, exosomal PD-L1 binds to PD-1 on the surface of T cells and TIM-4 has been shown to interact with phosphatidylserine on ABs, both driving immune suppression (5, 6).

In this review, we focus on EV responses in myeloid cells. Myeloid cells (e.g., macrophages, neutrophils, dendritic cells) are a key component of innate and adaptive immunity outnumbering other immune cells in most tissues. Importantly, myeloid cells can readily capture particulate materials and are key drivers and modulators of inflammation and tissue repair. Therefore, myeloid cells are likely highly responsive to EVs, driven by their receptor expression and/or capacity for phagocytosis and pinocytosis. Physiological context has become an important consideration in understanding EV responses, dictating which cargos are loaded into EVs as well as how recipient cells respond, including their capacity for uptake and stress or activation status (7). As such, EVs can serve opposing roles in autoimmunity and cancer depending on the inflammatory context. At steady-state innate cells capture EVs maintaining homeostasis and self-tolerance. In cancer, these mechanisms may function to dampen immune surveillance and promote chronic inflammation (8). EVs also play a progressive role as their systemic accumulation increases as we will later see in the example of metastasis. In autoimmunity, we see that the same EV cargo can promote the breakdown of immune tolerance driven by the inflammatory milieu.

Understanding how EVs drive physiological processes and identifying representative EV cargo signatures in pathological states is being utilized for therapeutic targeting and disease diagnosis. However, the role of EVs is only partially understood as the field of EV research is currently still in its infancy, and requires refinement in limitations of detection and isolation (1) as well as resolving contradictory results. The excitement around uncovering new biological mechanisms driven by these small particles, however, is generating rapid progress in the field. Here, we will examine what is known regarding mechanistic interactions between EV cargo and myeloid cells—defining a paradigm for understanding the mixed responses to EV cargo. We will also comment on the viability of therapeutic opportunities EVs generate, including inhibitory targeting of biogenesis and transfusion of EVs with an artificial therapeutic load and as vaccines.

## 2 EV Heterogeneity

There are currently three well described subtypes of EVs, differentiated by size and the cell processes responsible for their production. Apoptotic bodies are the largest and most studied class of EVs produced as a result of programmed cell death (2). Ectosomes and exosomes are smaller on average, jointly called small EVs (sEVs), and are produced by living cells. Differentiating the two sEVs, ectosomes are shed directly from the cell surface while exosomes (the smaller of the two sEV categories) originate from the cellular lumen bearing protein markers of their endocytic origin (9). The first EVs to be reported were ABs (50nm-5μm), as apoptosis was being closely studied in the 1960s (10). Microparticles (MPs, 50nm-1μm), also referred to as ectosomes, were first described in 1967 in the context of platelets and blood coagulation (11). The smallest EVs, exosomes (30-150nm) were first identified in the early 1980s (12, 13) in the study of transferrin receptor loss during reticulocyte maturation. These two sEV subtypes collectively were shown to be produced by healthy living cells of nearly all types—with EV dysfunction observed under cellular stress (14). The details of EV biogenesis have been recently reviewed elsewhere (3) and will not be covered here. Although the intracellular origin of the each EV subtype can be tracked, attempts to describe EV biogenesis and heterogeneity is confounded by overlapping size and biomarker criteria (2). Moreover, new classes of EVs are continually being identified including exophers (15), cytokine vesicles (16) and midbody remnants (17). The lack of consistent nomenclature in the field and biomarker oversimplification has driven pragmatic researchers to choose simpler terms like small and large EV to broadly understand biological functions.

### 2.1 Classical and Non-Classical EV Subtypes

**Figure 1:**
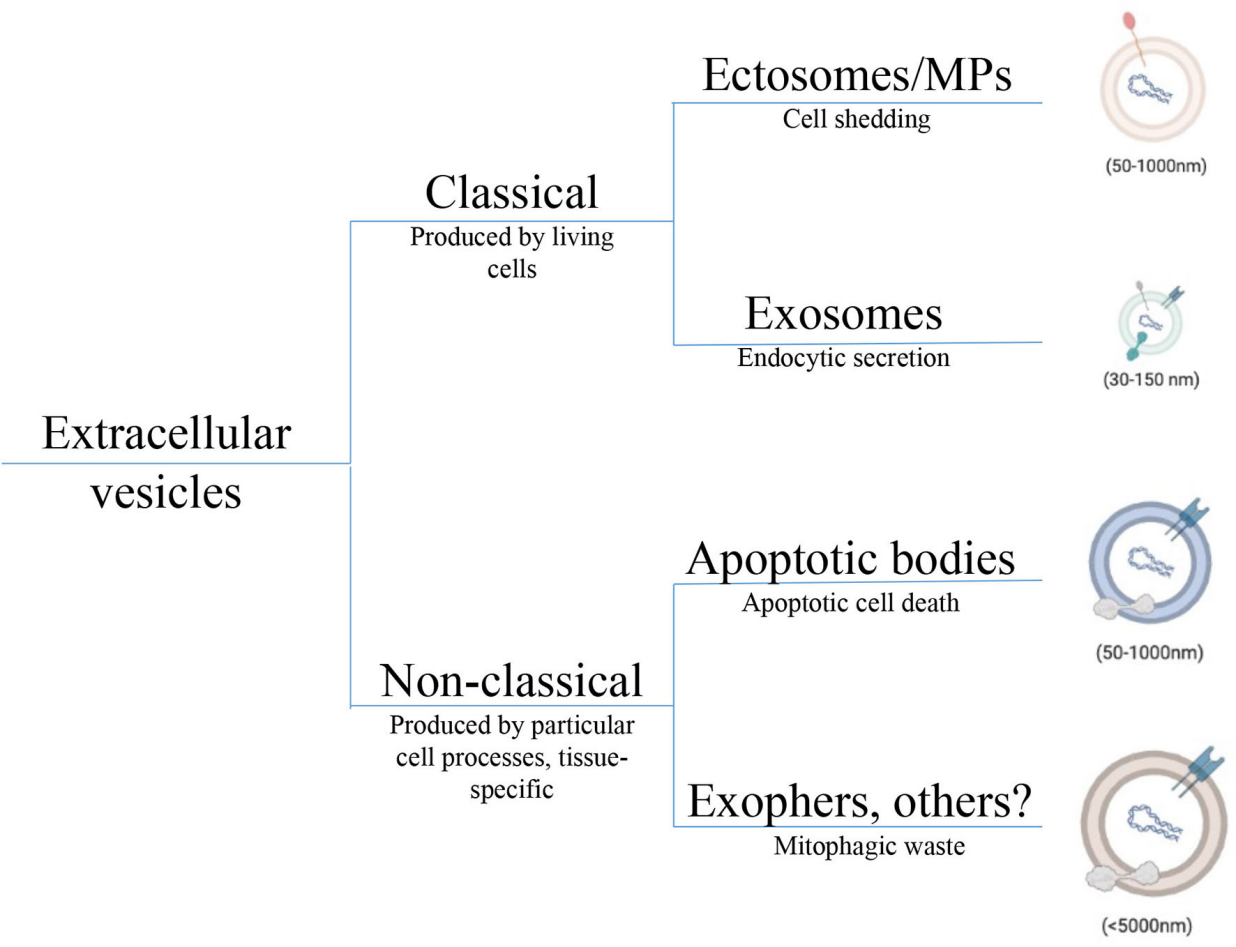
Taxonomic tree depicting EV heterogeneity. Diversity in EV populations can be grouped into classical and non-classical subtypes. Classical EVs are produced by health living cells; either shed from the cell surface as ectosomes, also called microparticles, or derived from inside the cell as exosomes. Although exosomes and ectosomes carry similar cargos including proteins, lipids, and nucleic acids, species of endocytic origin (e.g., HSPs, mtDNA) are only expected in exosomes. Non-classical EVs tend to be larger and correspond to a wide array of cell processes and include species like apoptotic bodies from dying cells, exophers released from neurons and cardiomyocytes as metabolic waste, and oncosomes derived from certain tumor cells. Non-classical subtyping allows for a better understanding of novel EVs that do not fit classical criteria preventing their misclassification. Cargos in each non-classical EV subtype are distinct but not fully understood. EV, Extracellular vesicle; HSP, Heat shock protein; mtDNA, Mitochondrial DNA.

Correctly characterizing and naming EVs is necessary for understanding each EV’s distinct physiological role. In a recent review, Kalluri and LeBleu streamline EVs into two categories, EVs shed from the plasma membrane of the cell as ectosomes, and EVs originating from intracellular compartments as exosomes (9). While this system works for such classical EVs, other particles must be included as non-classical subtypes (**Figure 1**). Specifically, this taxonomic classification of EVs does not fully capture EV heterogeneity, especially pertaining to large EVs. While some large EVs like oncosomes (<10μm), shed from the membrane of amoeboid tumor cells, fit into their system as ectosomes, ABs and exophers do not (18). ABs can neither be characterized as large or small EVs because of their wide size range nor as ectosomes or exosomes produced by live cells because they are formed out of complete cell components from dying cells. Also, the 4 μm exophers described recently in cardiomyocytes (15) and neurons (19), though containing intracellular mitochondrial components are too large to meet the exosome biogenesis criteria. Since exosome production involves formation of intraluminal vesicles (ILVs), exosomes are limited to ILV size between 30nm and 150nm (2). Further, Nicolás-Ávila et al. predict that production of exophers is related to autophagy dependant waste removal of abnormal mitochondria and protein, while exosomes are considered active agents of cell communication—although this has proven difficult to confirm. The authors also highlight that exophers are unrelated to ABs since apoptosis is not expected in quiescent cardiomyocytes or neurons. To enable accurate classification of such particles and to attribute responses correctly, we must include both exophers and ABs into the taxonomy of EVs. Inclusive criteria allow better resolution between particles, prevent misclassification, and can improve our understanding of complex EV responses.

To create consistency across different EV subtypes, the minimal information for studies of extracellular vesicles (MISEV) 2018 statement recommended the use thorough biomarker characterization of EVs (20). However, authors also acknowledge that defining EVs by biomarkers that reflect their biogenesis also leads to significant overlap between EV subtypes. Importantly, this limits tracking of EVs *in vivo* resulting in a lack of knowledge on rates of production and physiologically relevant EV concentrations. Consequently, most studies rely on *in vitro* derived EVs from a single cell or body fluid source used for treatment *in vitro* or for *in vivo* transfer. EV isolation methods rely on size- and density-based ultracentrifugation, size-exclusion columns, polyethylene glycol precipitation or microfluids-based immunoprecipitation, with each method yielding a partially pure EV population (1). Although small (s)EVs and be easily separated from large (l)EVs using these isolation methods, sEV isolation results in co-precipitation of other species in biofluid or media (16, 21). Heterogenous EV preparations confound our understanding of EV cargo signatures as well as cellular responses to EV treatment. More sophisticated isolation procedures, definitive biomarkers, basic biogenesis research, and nanoscale experimentation will be required for progress in this field. In the meantime, these limitations must be considered while studying the role EVs play in physiological processes.

## 3 Tumor EVs and Myeloid-Driven Inflammation

The heterogeneous cell populations in solid tumors and hematological malignancies produce a diversity of EV populations, changing by disease stage and therapeutic intervention. Understanding the diversity of EV populations within the tumor microenvironment (TME) as well as their nuanced effects on tumor pathology has been confounded by isolation and biomarker limitations. However, extensive characterization of immune responses to EVs in multiple cancer models have revealed the key role EVs play in chronic inflammation and immune tolerance. Innate immune cells are often abundant in the TME and express a wide array of receptors that capture or interact with EV particles suggesting that EVs regulate immunity by impacting innate immune function in the local tumor milieu. Supporting this prediction, studies have shown blocking EV production by tumor cells correlates with decreased overall immune responses in the TME (5) and increased myeloid cell infiltration, suppressive polarization, and differentiation (22–24) In this section, we will describe known molecular mechanisms of tumor EVs driven dysfunction in myeloid immune cells promoting chronic inflammation and immune tolerance in the tumor.

### 3.1 EV Dysfunction in the Tumor Microenvironment

Addressing the role EVs play in tumor pathophysiology begins with understanding the nature of EV production in the TME. While increased plasma EV concentration in cancer patients compared to healthy donors has been reported for many tumor types (25), production of EVs by the tumor and tumor-associated cells cannot be directly confirmed with current methodology. However, several studies suggest that tumor-associated cells produce higher levels of EVs with unique molecular signatures. EV biogenesis genes like Rab27 are upregulated in tumors and associated with poor prognosis (26). Further, hypoxia (27–30) and the cytokine milieu (e.g. IFNγ, TGFβ) in the TME modulate EV biogenesis and cargo loading (31). Basal levels of stress (7, 14) and therapeutic interventions like radiation (32) can also alter EV levels and composition. The normal response to stress *via* the tumor suppressor p53 was shown to drive exosome biogenesis *via* the TSAP6 protein, suggesting abnormal exosome production by p53 mutated tumor cells (33). Activating KRAS mutations, found in many different tumor types, have been shown to control miRNA loading into exosomes. McKenzie et al. found lower levels of *let-7a* miRNA packaged into exosomes when Ago2 is phosphorylated by the activated KRAS (34). *Let-7a* miRNA has been show to target KRAS with decreased levels been reported in KRAS and BRAF tumors (35). Furthermore, high levels of *let-7a* were shown to promote anti-tumor microglia activation (36), suggesting a role for exosomes on innate myeloid cell responses. Together, tumor mutations and TME stimuli may drive a unique EV signature impacting systemic and local tumor immune responses.

### 3.2 EVs and the Tumor Wound Hypothesis

Tumor promoting inflammation and immune suppression in the TME has been understood by drawing parallels between the tumor and a healing wound (37). In the “tumor wound” concept, the TME hijacks wound healing mechanisms driving immunosuppressive and reparative stages of inflammation without resolution stage, resulting in a chronic wound-like state. In injured tissue damaged cells release a wide range of damage-associate molecular patterns (DAMPs) including heat shock proteins (HSPs), Glypicans, HMGB1, mitochondrial DNA (mtDNA) driving the production of alarmins (IL-1, IL-33) and other effectors that recruit and activate immune effectors *via* pattern-recognition receptors (PRRs) (38). In the TME, tumor-derived DAMPs can include mitochondrial components released due to the Warburg effect, miRNA and HSPs released due to genetic mutations and other factors like hypoxia. These DAMPs have been widely reported in tumor EVs across many tumor models (**Table 1**) and can drive inflammation in the tumor. In the healing wound, other signals from the unvascularized repairing tissue, hypoxia for example, drive a switch from acute inflammation to tolerance, where alternatively activated myeloid cells promote the immunosuppressive, pro-angiogenic and fibrotic stages of wound healing. A growing tumor is similarly supported by the suppression of anti-tumor immunity, angiogenesis and fibrosis. The mechanisms that sustain tumor growth in an unresolving state are not fully understood. However, the continuous exposure to EV-DAMPs to myeloid cells poised for particle capture, may contribute to tumor promoting inflammation. The signalling pathways stimulated upon EV exposure point to a key role of EV-mediated induction of tumor promoting inflammation.

**Table 1:** Examples of EV cargo effects on signalling in myeloid cells.

Cargo	Species	Function on myeloid immune cells
DNA	gDNA Micronuclei (39)	Cytoplasmic STING activation in dendritic cells
	mitoDNA (40)	Endosomal TLR9-mediated suppressive macrophage polarization
RNA	miRNA (41)	STAT3-mediated MDSC activation
	Y-RNA (42)	TLR7 mediated PDL-1 upregulation in monocytes
	dsRNA (43)	TLR3-mediated neutrophil recruitment at metastatic sites
	lncRNA-HOTAIRM1 (44)	MDSC expansion *via* STAT3
Mitochondria	Cardiac autophagy (15)	Phagocytic clearance by macrophage supports tissue homeostasis
Lipids	Phosphatidylserine (45)	Receptor-mediated regulatory macrophage polarization
Cytokines	TGF-β1 (46)	Dendritic cell driven T cell suppression
Self-antigen	MART1 (47)	Delivery of tumor antigen to activated dendritic cells
Checkpoint molecules	PDL1 (5)	Delivery of PDL1 to myeloid cells leads to systemic T cell exhaustion
Integrins	Tissue specific integrin signature (48)	Integrins prime Kuppfer cells for liver metastasis
Microbiome components	Gram negative cell wall components (49)	TLR-4 ligands in bacterial EVs activate innate immune cells

#### 3.2.1 Vesicular TLR-Ligand Driven Inflammation

Myeloid cells express a battery of pattern-recognition receptors including Toll-like receptors (TLR), retinoic-acid inducible gene (RIG-I), and stimulator of interferon genes (STING) that are highly responsive to DAMPs allowing rapid immune reactivity to tissue injury (50). In the tumor, EV-DAMPs have been shown to engage both plasma membrane-localized TLRs (e.g., TLR2 and TLR4) and endosomal TLRs (e.g. TLR3, 7, and 9) driving potent responses *via* the transcription factor, NFκB (51). In breast cancer, palmitoylated proteins in tumor EVs can act as TLR2 ligands leading to upregulation of proinflammatory cytokines and chemokines including CCL2, IL-6 and GCSF in macrophages (52). TLR2 ligand, HMGB-1 was also found in lung cancer and shown to drive NF-κB-dependent metabolic reprogramming of macrophages at metastatic sites (53). Also using a lung cancer model, Fabbri et al. demonstrated that exosomal miRNAs (i.e. miR-21 and miR-29a) induce IL-6 and TNFα in macrophages *via* a TLR7-NFκB dependent mechanism promoting inflammation and metastasis (54). Similarly, exosomal RNA can activate TLR3 promoting neutrophil recruitment at metastatic sites *via* induced expression of CXCL chemokines (43). To offer further insight into which RNA species are EV associated, an atlas of vesicular and non-vesicular RNA from healthy biofluids was recently published (55). In pancreatic cancer, sEVs from were shown to carry genomic dsDNA (56) that act as a TLR-9 ligand activating myeloid cells (57). However, Jeppesen et al. showed that genomic dsDNA-TLR9 ligands are secreted from tumor cells independent of exosomes, while HSP-TLR2 and RNA-TLR7 ligands are enriched in sEVs (21). Jeppesen et al. argue that genomic DNA (gDNA) does not exist in the same subcellular location for endosomal loading required for exosome biogenesis. However, gDNA may become exosome associated, adhering to positively charged EVs prior to capture by donor cells. The presence of double stranded mitochondrial DNA (mtDNA) is less controversial due to subcellular location. MtDNA has been observed in breast and prostate cancer EVs at higher concentrations that noncancer epithelia (58). Although transfer of mt-DNA between cell types is likely EV-mediated, most researchers have studied the role of mt-DNA alone. Mt-DNA has been shown to induce TLR9-mediated NF-κB activation driving regulatory polarization in macrophages in liver cancer (59) as well as activation of neutrophils in various forms of injury (60). Further, non-immune cancer-associated fibroblasts were also shown respond to mt-DNA *via* TLR-9 contributing to therapeutic resistance to taxanes (40). In addition to inflammatory modulation, mutations in tumor mt-DNA can also regulate mitochondrial metabolism in recipient cells (61) but have not been well-studied in the context of myeloid immunity. Size-based EV isolation procedures leading to the co-precipitation of non-vesicular nucleic acids and other molecules will confound data on EV-mediated gene induction until more specific isolation methods are found.

DAMP-TLR activation in myeloid cells can drive both pro-inflammatory and regulatory responses *via* mechanisms that are not fully understood. One hypothesis is that pro-inflammatory effectors are expected to drive pleiotropic effects on immune cells and other cells of the body, supporting both inflammatory and suppressive responses in a context dependent manner. In the context of macrophages in chronic conditions, experts have encouraged a spectrum polarization model over the dichotomous M1-M2 model, which better describes acute conditions. This integrative spectrum model also allows for a better understanding of chronic inflammation driven by tumor EVs. Interestingly, EV studies described here show upregulation of both pro- and anti-inflammatory cytokines and effectors in myeloid cells pointing to concomitant responses to mixed EV cargo. Secondly, chronic exposure to TLR ligands drives negative feedback mechanisms that lead to reduced inflammatory cytokine and increased regulatory cytokine production. Repeat treatments with DNA drive tolerance in macrophages *via* TLR-9 as seen by reduced TNFα production (62), mimicking the chronic nature of EV responses. This chronic exposure to TLR ligands is also expected to dictate hematopoietic outcomes starting in the bone marrow, driving a myeloid differentiation bias leading to reduced lymphoid to myeloid ratios in blood (63), with trained immunity being a common feature of aging and cancer pathophysiology (64).

#### 3.2.2 STAT3 Driven Inflammation

Upon TLR activation of NFκB transcriptional program a further activation of STAT3 is driven by autocrine IL-6 stimulation, in turn amplifying the overall inflammatory response (65). Chalmin et al. demonstrated the TLR2 ligand Hsp70, found on the exosome surface, activates STAT3 in MDSCs *via* autocrine action of IL-6 (66). These activated MDSCs produce IL-10 and upregulate arginase 1 activity inhibiting T cell proliferation. The EV-driven IL-6-STAT3 axis was also shown to keep bone marrow precursors in an immature state inhibiting the differentiation to mature DCs capable of anti-tumor responses (24). A STAT3 regulatory signature was also observed in monocytes treated with glioblastoma exosomes correlating with increased interferon-ɣ production (67). A similar TLR2/4-STAT3 response driven by HSPs was seen in bone marrow-derived dendritic cells (BMDCs) promoting a pro-tumor IL-6, PGE-2, IL-1, and TNF response after exosome treatment (68).

EVs have also been shown to control the NFκB-STAT3 axis *via* the action of specific miRNA cargo. Both NFκB-driven and exosomal *miR-21a* was shown to silence PDCD4, a tumor suppressor and IL-6 inhibitor, promoting the expansion of MDSCs in a IL-6/STAT3 dependant manner in lung cancer (69). Exosomal *miR-106b* found in colorectal cancer also suppresses PDCD4 driving both IL-6/STAT3 and mTOR signalling to promoting regulatory polarization in tumor macrophages (70). The exosomal *miR-222-3p* in ovarian cancer, has been shown to silence SOCS3 in monocytes promoting a STAT3-mediated regulatory signature in macrophages including Arg1 and CD206 expression (71). It is not clear whether the concentration of miRNA in sEVs can reach a local concentration capable of eliciting an immune response (72) or whether tumor derived sEVs may be continuously produced eliciting a cumulative response over time.

### 3.3 EVs and Pre-Metastatic Niche Formation

EV-macrophage interactions have been implicated in the priming of the metastatic niche in several studies. In Hoshino et al., exosomes from tumor cell lines with a liver metastatic ability were compared to non-metastatic lines and were shown to carry integrin α_v_β_5._ This allowed preferential binding to liver-specific cells including CD169^+^ Kupffer macrophages, driving an inflammatory phenotype *via* integrin-mediated Src phosphorylation and upregulation of s100 and fibronectin genes (48). Similarly, in Costa-Silva et al., migration inhibitory factor (MIF) found in pancreatic cancer exosomes was taken up by Kupffer macrophages driving TGFβ and fibronectin production priming the liver for metastasis (73). In Peinado et al., the receptor tyrosine kinase MET found in melanoma exosomes was captured by bone marrow progenitors driving vasculogenesis and promoting metastasis in a systemic fashion (74). Another group studying brain metastasis, showed that *miR-19a* carried from astrocyte exosomes to tumor cells led to reduction of PTEN and increased CCL2 *via* NFκB in tumor cells driving suppressive myeloid infiltration (75). Interestingly, injection of melanoma EVs drove downregulation of IFNAR1 in monocytes at distant sites driving lung metastasis *via* fibronectin deposition (76). This downregulation of IFNAR was thought to dependent on p38 expression driven by exosomal mRNA mediated TLR3-NFκB activation (43, 76). Albeit *via* differing mechanisms, these studies all support the pathogenic role of distant EV-mediated cell-cell communication in tumor metastasis.

### 3.4 Modulating the Interferon Response

Activation of TLRs in myeloid cells *via* DAMPs drives both NFκB and interferon regulatory factor (IRF) signalling. However, several studies suggest tumor EVs may inhibit interferon (IFN) expression and IFN responses. In an elegant approach, Gao et al. injected tumor derived exosomes into mice infected with both DNA and RNA viruses to examine EV-driven immune suppression. The data showed that exosome-delivered epidermal growth factor receptor (EGFR) reduced INFβ expression in macrophages after viral infection (77). Mechanistically, this was the result of tumor EV-EGFR driven Mitogen-Activated Protein Kinase Kinase Kinase 2 (MEKK2) phosphorylation, inhibiting IRF3 dimerization and IFN production. Similarly, hepatocarcinoma EVs containing interferon induced transmembrane Protein 2 (IFITM2) reduced IFNα production by HBV infected dendritic cells (78) while exosome delivered IRF2 (IRF2 is a repressor of Type I IFN signaling) limited IFNα/β production in macrophages. EVs can also directly reduce cellular responsiveness to IFN stimulation. For example, melanoma EVs can directly down-regulate interferon alpha and beta receptor 1 (IFNAR1) in myeloid cells *via* p38 activation dampening responsiveness to IFN stimulation (76). IFNAR downregulation was also required for maintaining suppressive activation in MDSCs (79). Thus, cumulatively the data suggests EV exposure can have wide ranging impact on IFN response influencing initial stimulation and downstream IFN-induced transcriptional programs.

EVs produced by tumors also contain self-associated molecular patterns (SAMPs) that potentially modulate tumor inflammation by molecular pathways that are distinct from those described above (80). The siglec-family self-pattern recognition receptors (SPRRs) bind to self-sialic acid residues abundant on EVs (81). Hypersialyation has been reported in both solid tumors and hematological malignancies (82) with sialic acid enrichment on tumor EVs (83). These surface glycans are required for EV internalization by many cell types (83). For example, CD169 (also known as Siglec-1 or sialoadhesin) is an exosome endocytic receptor for macrophages by binding α2,3-linked sialic acids (84). CD169 is expressed on sentinel macrophages at key interfaces in the body (blood-spleen, fetal-maternal, lung-air, etc). In peripheral lymphoid organs, CD169^+^ macrophages play a key role in orchestrating the response to particulate antigens including ABs and virus particles (85–87). Infection with vesicular stomatitis virus (VSV) induced CD169 upregulation and recruitment of a DAP12/SHP2/TRIM27 complex (88). This inhibitory complex ubiquitinated and degraded TBK1, inhibiting IRF3 phosphorylation and downregulating the type I IFN response (88). Because CD169 is an IFN stimulated gene (ISG) this mechanism may be a negative feedback loop, promoting tolerance to self-sialic acids. Supporting this concept, in a mouse model of HIV, CD169 deletion lead to better control of viral load with increased IFN-I production (86). Other siglec family receptors (e.g. Siglec-H, Siglec-G and Siglec-10) also contain immunoreceptor tyrosine-based inhibitory motifs (ITIMs) or recruit adaptor proteins with ubiquitinase activity, with the exception of CD33 and Siglec-H which have activating function (82). Binding of EV sialic acids to siglecs may similarly attenuate IFN responses, modifying the overall effect of EV-DAMP signalling *via* TLR (89, 90). Specifically, myeloid siglec-7 and siglec-9 have been shown to reduce HLA-DR and CD86 expression upon engagement with tumor-derived sialic acids promoting T cell suppression in the pancreatic TME (91). Siglec^+^ macrophages were also found in the bone marrow, controlling HSC (92) and erythrocyte egress (93).

**Figure 2:**
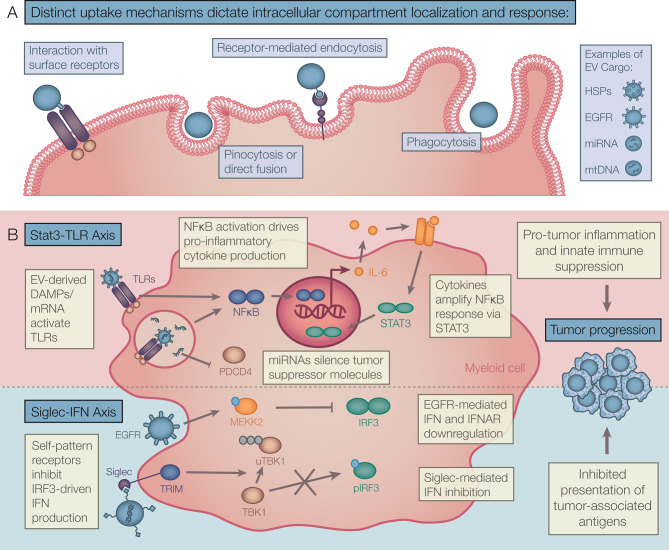
Theoretical framework for tumor EV driven myeloid responses. **(A)** EV capture mechanisms include binding directly to cell surface receptors, direct fusion, and internalization by pinocytosis, receptor-mediated endocytosis, and phagocytosis. Distinct capture mechanisms deliver EV cargo to compartment-specific receptors driving diverse EV responses in recipient myeloid cells. **(B)** A range of tumor EV cargos act together to drive tumor promoting inflammation. Chronic interaction with EV-DAMPs and other effectors activate NF-κB and STAT3 signalling resulting in accumulation of late-stage cytokines. Also, self-molecular patterns and other effectors found in EVs attenuate type I IFN production and/or response. These signals can modulate acute inflammation and presentation of tumor antigens. EV, Extracellular vesicle; DAMPs, Danger-associated molecular patterns; NF-κB, Nuclear factor Kappa B; STAT, Signal transducer and activator of transcription; IFN, Interferon.

These studies collectively show that EVs package both DAMPs and other endogenous immunity-inducing structures driving diverse responses in myeloid immune cells (**Figure 2**). While one signal from DAMPs provides activation of TLR-STAT-NFκB pathways resulting in inflammatory cytokine production, a second signal from self-patterns and other cargo inhibits the IFN-I response attenuating inflammation and self-antigen presentation. This results in a unique EV response that promotes the breakdown of immune surveillance in cancer and prevents autoimmunity under homeostatic conditions.

## 4 Autoimmune EVs Promote Inflammation and Tissue Destruction

In cancer studies the popularity of the term “exosome” has resulted in its use as a generic descriptor of EVs despite its specific endocytic definition that precludes other EVs (94). In the field of autoimmunity, outside of apoptotic bodies that have been widely studied, the term microparticle (MP) is more prevalent in the study of sEVs. Because *in vitro* derived MPs mainly use lower centrifugation speeds, these studies capture larger vesicles, enriching the ectosomal or cell shedding EV phenotype. MPs also lack endocytic markers like chaperone HSPs and are enriched in phosphatidylserine (95). Exosomes, have also been studied in autoimmunity, however, the role they play distinct from other sEV subtypes is not clear.

Many of the same EV cargo components have been found in cancer and autoimmunity. Similar to observations in cancer EVs, higher levels of EVs have been reported in Sjögren’s syndrome, systemic lupus erythematosus (SLE) and rheumatoid arthritis (RA) compared to healthy individuals (96, 97). Here too, the presence of DAMPs in EVs drives inflammation in a TLR-dependent manner. The focus in autoimmune disease has been on the TLR7 and TLR9 ligands DNA and RNA which are abundant in sEVs and apoptotic bodies found at high concentrations in circulation (98). We have previously reviewed the systemic effects of ABs in SLE (99), and will focus on the role of sEVs in this review. Exosomal *miR-let-7b* found in RA synovial fluid, promoted TLR7 activity in myeloid cells of inflamed joints, stimulating production of IL-1β and IL-6 (100). Further, removal of DNA from EVs by circulating DNASE1L3 prevents autoimmunity in healthy mice, while in lupus, DNASE1L3 null mutations and the presence of anti-DNA autoantibodies protects against DNA degradation promoting inflammation in a TLR-MyD88-dependent, STING-independent mechanism (101). HMGB1 has also been found in autoimmune EVs and functions to promote inflammation *via* TLR4 in myeloid cells (102). In these studies, sources of EVs were varied, arising from platelets, endothelial cells, fibroblasts, and dying lymphocytes. A similar mechanism was also observed in scleroderma (103). EVs were also found as immune-complexes (ICs) with the anti-DNA/RNA autoantibodies characteristic of RA and SLE, promoting complement-driven, TLR-mediated DC inflammatory activation (104). EVs were also shown to contain citrullinated self-proteins contributing to the formation of inflammatory immune complexes in SLE and RA, driving monocyte and macrophage activation (105, 106). In lupus, ICs were required for a metabolic switch to glycolysis in macrophages leading to production of IL1 and ROS (107), further exasperating autoimmunity.

In type 1 diabetes (T1D) pancreatic β-cell specific destruction is triggered, rather than the systemic tissue destruction seen in SLE. Because EV release occurs prior to immune β-cell destruction, EVs may play a role in disease initiation. Using *in vitro* derived EVs from MSC-like cells from the NOD pancreas, Rahman et al. show that the intrinsic endoplasmic reticulum stress in cells of the pre-diabetic pancreas controls EV cargo driving DC-mediated priming of autoreactive T and B cells *via* IFNγ upon EV treatment (108). Moreover, the T1D autoantigens glutamic acid decarboxylase 65 (GAD65), zinc transporter 8 (ZnT8), and β-cell resident glucose transporter 2 (Glut2) were found within T1D islet EVs (109), supporting both the EV delivery of self-antigen as well as auto-antibody-EV immune complex driven myeloid cell activation *via* F_c_ receptors. Micro RNA species have also shown to play a key role in T1D pathogenesis. In addition to targeting many metabolic genes, the exosomal *miR-29* derived from β-cells, also found in tumor exosomes, also induces TLR7-MyD88 dependent inflammatory cytokine production, including IFN-I responses (110). Exosomal *miR-29* has also been found at higher levels in type 2 diabetes, driving metabolic reprogramming of macrophages *via* TRAF3 promoting systemic insulin resistance (111). Like observations in cancer and systemic autoimmune diseases, EVs appear to largely promote inflammation in T1D. However, EV-driven protection from autoimmunity has also been reported. AhR ligands found in EVs and other endogenous sources, attenuate autoimmunity in cases of EAE and lupus (98, 112).

The inflammatory context in autoimmunity and cancer lead to myeloid responses to the same EV cargo to both break tolerance and suppress immune responses *via* mechanisms that remain elusive. Studies on differences in EV concentration and IFN induction by autoimmune EVs versus tumor EVs may offer insights into how this is achieved.

## 5 EVs and Therapeutic Opportunities

The most readily apparent application for EVs is their potential as diagnostic tools. Since they contain biomarkers of cell status, EVs from blood, ascites, and urine can potentially be utilized to serve as a liquid biopsy diagnostic differentiating healthy from diseased states. Targets including metabolic peptides, nucleotide species including circulating tumor (ct)DNA, autoantibody immune complexes and microbiome-derived EV cargo are being actively explored for diagnostic purposes (25, 113). Though finding EV biomarkers that differentiate related disease states have proven difficult, this line of investigation may be useful for both cancer and systemic autoimmune conditions (114, 115). A thorough review of EV cargo biomarkers being investigated for diagnostic application can be found elsewhere (116).

As detailed in this review, EVs promote inflammation and disease pathophysiology by variety of different mechanisms. Therefore, the inhibition of EV biogenesis using small molecules agents is being explored as a therapeutic opportunity in both cancer and autoimmunity. Small molecules including GW4869 and dimethyl amerlioride, as well as siRNA against proteins in EV biogenesis pathways, have been used extensively for research and hold promise in targeting pro-tumor inflammation driven by EV-DAMPs. However, each of these molecules have exhibited off-target effects on cellular physiology; for example, GW4869 which targets neutral sphingomyelinase (N-SMase) can also have effects on autophagy (117). Datta et al. have validated novel exosome inhibitors using prostate cancer cells, identifying natural small molecules like Forskolin and antibiotics like Manumycin A as blockers of exosome biogenesis (118). Rab-GTPase inhibitors, along with other anti-tumor functions on tumor growth and cytokine secretion, can also antagonize EV biogenesis and can be used therapeutically (119). The design and delivery of siRNA against proteins involved in biogenesis like Rab27a/b in the case of exosomes may allow for more specific targeting than small molecule inhibitors (120). However, to identify the best therapeutic target with the fewest off-target effects, a more comprehensive understanding of both EV responses and biogenesis pathways is required.

The targeting of EV capture and response by myeloid immune cells is another strategy to attenuate chronic inflammation in both cancer and autoimmunity. As described earlier, the siglec-sialic acid axis may antagonize IFN-mediated tumor destruction. Several anti-siglec antibodies are being investigated including anti-siglec-7 and anti-siglec-33 which target NK cells and immature myeloid cells respectively attenuating their regulatory function (121). Targeting other siglecs and scavenger receptors may lead to the abrogation of EV capture or facilitate delivery of therapeutical active EV cargo. EV-CD169 interactions in macrophages also drive antigen presentation in the context of infection and chemotherapy (86, 87). Capitalizing on this observation, Edgar et al. show that liposomes containing antigens targeted to CD169 *via* decoration with sialic acid residues induces CD4 T cell responses, but requires liposome loading with high-doses of TLR7-IFN promoting adjuvant for cytolytic CD8 T cell responses (122). The myeloid responses to EV-DAMPs can be inhibited *via* the targeting of the TLR-NFκB pathways. Though this is promising in autoimmunity, in cancer TLR agonism is being investigated in the clinic due to its ability to promote tolerance to the tumor (123). However, TLR4 driven myeloid tolerance has also been blamed for paclitaxel resistance which acts as a TLR4 agonist (124). Inhibiting this pathway in cancer may involve timing where, as a neoadjuvant strategy, EV-DAMP driven NFκB activation can be inhibited temporarily prior to T cell activation or chemotherapy. Lastly, to promote MHC loading of tumor neoantigen in APCs after EV capture, an interesting recycling regulator Rab17 (shown to prevent presentation of AB derived self-antigens) could be targeted to promote tumor-specific immune destruction (125). Because EV-myeloid cell interactions are also part of homeostatic processes, targeting EV biogenesis, capture and responses to EVs may drive off-target effects that remain to be fully understood in *in vivo* disease contexts.

Furthermore, the administration of bioactive EVs is being investigated in various therapeutic strategies. The use of EVs for delivery is advantageous over liposomes with the ability to easily disguise as self in the body. Moreover, EVs decorated with integrins can target specific tissue sites allowing for specific delivery of therapeutic cargo (48). In mice, exosomes loaded with IL-12 (exoIL2) were shown to promote an antitumor T cell response, superior to the recombinant cytokine alone due to the improved pharmacokinetics (126). Because IFN signalling in APCs drives antigen presentation and cross presentation to both CD8+ and CD4+ T cells (127), therapeutic administration of large doses of EVs loaded with STING agonist dinucleotides (exoSTING) is being explored to promote adaptive anti-tumor immune responses. Jang et al. showed that exoSTING exerts tumor control by targeting APCs preferentially which induces antitumor T cell responses with long-term memory T cell induction (128). Although STING and RIG-I activation has been reported in dendritic cells, and other cells stimulated by EVs (129, 130), it may be attenuated downstream by Siglec-TBK1-dependant mechanisms *in vivo*. Limiting IFN-driven antigen presentation in response to DAMP-containing EVs may be an evolutionary mechanism to limit excessive self-peptide presentation and autoimmunity, also serving to limit tumor neoantigen presentation. Modulating the dose of EVs and additional modifications to such therapeutically administered EVs, like sialidase treatment might circumvent regulatory immune responses. In autoimmunity, these administered EVs can functions as decoys to autoantibodies. Casella et al. showed that EVs from oligodendrocytes contain myelin and related antigens and can be used to subvert myelin destruction in a model of multiple sclerosis (131). The applications for decorating or loading EVs with many different cell-targeting or immunomodulatory agents suggest EVs could be utilized as versatile payload delivery agents.

EVs have also been explored as cell-free anti-tumor vaccines. Specifically, vaccination with exosomes derived from tumor antigen loaded DCs reduced tumor burden by induction of anti-tumor T cell responses (132). DC-derived sEVs can carry whole tumor associated antigens (TAAs), TAA peptide-loaded MHC/HLA, and co-stimulatory signals significantly improving vaccination efficacy compared to whole tumor lysate vaccination (133). However, the requirement of MHC molecules for functionality/immune responses to DC-derived EVs (DEX) is not clear. For example, Hiltbrunner et al. reported that whole antigen in the absence of MHCI and II is sufficient to induce a DEX-mediated T cell response suggesting internalization and antigen processing are the key relevant components for EV-driven immune responses (134). Supporting this prediction, DEX loaded with antigen can drive stronger *in vivo* T cell responses than DC-EVs from an ectosomal origin, suggesting an involvement of the endocytic compartment in the efficacy of DEX (135). The clinical success of DEX as vaccines will likely require large, non-physiological doses of EV particles which necessitated the large-scale culture of donor-matched DCs potentially limiting this approach. In contrast to DEX, the application of the more readily available tumor EVs is currently limited to DC vaccines primed with tumor EVs. Andre et al. show that exosomes isolated from melanoma ascites contain TAAs like MART1 and gp100, and when used to stimulate donor-matched dendritic cells promote antigen-specific T cell activation and cytolytic function *in vitro* (47). Further. the *in vivo* injection of DCs treated with *in vitro* tumor cell-derived EVs into tumor-bearing mice was reported to drive tumor rejection in a T cell-dependent manner (136). These DC vaccine strategies are similarly challenged with the culture and dosing of DCs at therapeutic concentration as well as HLA-matching of both EVs and DCs required to induce the desired anti-tumor response. The pre-dominantly immunosuppressive role for tumor EVs is supported by a large body of literature and suggests the direct use of tumor-derived exosomes as TAA containing vaccines may not be a readily applicable approach given our current level of understanding.

## 6 Conclusion

Extracellular vesicles take on a wide heterogeneity of subtypes and relay a specific molecular signature from their cell of origin. The effects of a diverse EV cargo on myeloid immune cells are revealing their role in inflammation and diseases of immune dysregulation including cancer and autoimmunity. In the tumor, context dependant mechanisms drive a pro-tumor inflammation both locally and at metastatic sites promoting a breakdown of immune surveillance, whereas in autoimmunity, mechanistically similar EV signals promote a breakdown of tolerance and autoimmune pathology. Importantly, EVs are potent modulators of the IFN response which may provide protective function preventing autoimmunity, but also provide a significant barrier to anti-cancer immunity. EVs are more biologically complex compared to other cell-cell communication systems (e.g., cytokines) and this complexity has provided a barrier to our understanding, but also opportunities to harness EV biology for therapy. In this vein, the clinical application of EVs is numerous including disease diagnosis/prognosis, engineered delivery of therapeutic cargo, and administration of anti-tumor vaccines. Ultimately, these therapeutic strategies hold great promise but require much more research for safe and effective execution. In the final analysis, although great strides towards the understanding of EVs have been made, this burgeoning field promises to reveal novel cellular mechanisms involved in health and disease.

## Author Contributions

PM and TM conceived the manuscript. All authors contributed to the article and approved the submitted version.

## Conflict of Interest

The authors declare that the research was conducted in the absence of any commercial or financial relationships that could be construed as a potential conflict of interest.

## Publisher’s Note

All claims expressed in this article are solely those of the authors and do not necessarily represent those of their affiliated organizations, or those of the publisher, the editors and the reviewers. Any product that may be evaluated in this article, or claim that may be made by its manufacturer, is not guaranteed or endorsed by the publisher.
